# Towards standardized postprocessing of global longitudinal strain by feature tracking – OptiStrain CMR-FT study

**DOI:** 10.1186/s12872-019-1255-4

**Published:** 2019-11-27

**Authors:** Robert Heinke, Faraz Pathan, Melanie Le, Tommaso D’Angelo, Lea Winau, Christophe Arendt, Thomas J. Vogl, Andreas Zeiher, Eike Nagel, Valentina O. Puntmann

**Affiliations:** 1 Institute of Experimental and Translational Cardiac Imaging DZHK Centre for Cardiovascular Imaging Goethe University Hospital Frankfurt, Theodor-Stern Kai 7, 60590 Frankfurt am Main, Germany; 2 Department of Cardiovascular Imaging, Menzies Institute for Medical Research, Hobart, Tasmania Australia; 3 Department of Biomedical Sciences and Morphological and Functional Imaging, G. Martino University Hospital Messina, Messina, Italy; 4 Department of Radiology, Goethe University Hospital Frankfurt, Frankfurt-am Main, Germany; 5 Department of Cardiology, Goethe University Hospital Frankfurt, Frankfurt-am Main, Germany

**Keywords:** Feature tracking, Myocardial deformation, Longitudinal strain, Cardiac magnetic resonance, Standardization

## Abstract

**Background:**

Left ventricular global longitudinal strain (GLS) with cardiovascular magnetic resonance (CMR) is an important prognostic biomarker. Its everyday clinical use is limited due to methodological and postprocessing diversity among the users and vendors. Standardization of postprocessing approaches may reduce the random operator-dependent variability, allowing for comparability of measurements despite the systematic vendor-related differences.

**Methods:**

We investigated the random component of variability in GLS measurements by optimization steps which incrementally improved observer reproducibility and agreement. Cine images in two-, three- and four-chamber-views were serially analysed by two independent observers using two different CMR-FT softwares. The disparity of outcomes after each series was systematically assessed after a number of stepwise adjustments which were shown to significantly reduce the inter-observer and intervendor bias, resulting standardized postprocessing approach. The final analysis was performed in 44 subjects (ischaemic heart disease *n* = 15, non-ischaemic dilated cardiomyopathy, *n* = 19, healthy controls, *n* = 10). All measurements were performed blind to the underlying group allocation and previous measurements. Inter- and intra-observer variability were tested using Bland-Altman analyses, intra-class correlation coefficients (ICCs) and coefficients of variation (CVs).

**Results:**

Compared to controls, mean GLS was significantly lower in patients, as well as between the two subgroups (*p* < 0.01). These differences were accentuated by standardization procedures, with significant increase in Cohen’s D and AUCs. The benefit of standardization was also evident through improved CV and ICC agreements between observers and the two vendors. Initial intra-observer variability CVs for GLS parameters were 7.6 and 4.6%, inter-observer variability CVs were 11 and 4.7%, for the two vendors, respectively. After standardization, intra- and interobserver variability CVs were 3.1 and 4.3%, and 5.2 and 4.4%, respectively.

**Conclusion:**

Standardization of GLS postprocessing helps to reduce the random component of variability, introduced by inconsistencies of and between observers, and also intervendor variability, but not the systematic inter-vendor bias due to differences in image processing algorithms. Standardization of GLS measurements is an essential step in ensuring the reliable quantification of myocardial deformation, and implementation of CMR-FT in clinical routine.

## Background

Global longitudinal strain (GLS) is an important prognostic biomarker in the evaluation of the left ventricular (LV) function [1–3]. Visual assessment of wall motion abnormalities is fast but dependent on the experience of the observer and short of objective quantification [4, 5]. In echocardiography, GLS derived by automated speckle-tracking has been shown superior in detecting and quantifying subtle impairment of LV systolic function [2], as well as to provide higher predictive value for mortality than LV ejection fraction (LV-EF) in the presence of regional wall motion abnormalities [1], or isolated GLS reduction in the presence of global (diffuse) systolic impairment [3].

Feature tracking (FT) by cardiac magnetic resonance (CMR), or CMR-FT, has conceptual similarities with speckle tracking, in providing quantitative assessment of myocardial deformation [6]. Despite the methodological differences in image acquisition and postprocessing, the similarity extends to the use of routinely acquired cine (steady-state free precession, SSFP) images, avoiding the need for additional dedicated sequences, such as tagging [7, 8]. Numerous studies reported on validation and agreement with other deformation techniques (reviewed elsewhere ([8, 9]), which, in summary, reveal that the measurements derived by CMR-FT are not easily transferrable, nor in scale or precision. One of the reasons for the differences is a number of methodologically different software solutions, pertaining numerous approaches to automatic contour-placement and tracking, as well as underlying algorithms of strain calculation [7]. Moreover, despite high reproducibility GLS in some single centre studies, there remains a remarkable inter-centre difference despite the use of same vendor, reflecting an important source of operator induced, or random variability [10, 11]. We hypothesized that standardization of user postprocessing may reduce the random component of variability. Improved precision of measurements may support transferability of CMR-FT and allow comparability of intervendor and intercentre results, despite the systematic differences, owing to the different image processing algorithms used by vendors. In this study, we undertook a systematic analysis and elimination of potential errors to guide the development of standardized operating procedure for local reads by comparison of two different vendors.

## Methods

Anonymized datasets were sourced from the prospective longitudinal observational multicentre investigator-led study [12–14]. Groups of unrelated subjects with either known ischaemic heart disease (IHD) or non-ischaemic dilated cardiomyopathy (NIDCM), were composed to examine the influence of regional wall motion abnormalities (RWMA) and diffuse myocardial impairment on postprocessing of GLS, respectively. The control group consists of subjects with normal blood pressure, low-pretest likelihood of cardiomyopathy, normal LV mass, volumes, and global systolic function, as well as absence of myocardial scar on late gadolinium enhancement (LGE) imaging and no regular medication. Clinical meta-data, including systolic/diastolic blood pressure (BP), body mass index (BMI) were recorded. Exclusion criteria for all subjects were the generally accepted contraindications to CMR (implantable non-MR safe devices, cerebral aneurysm clips, cochlear implants).

### CMR image analysis and acquisition

The CMR protocol, details of image acquisition and postprocessing have been reported previously [12–14]. All subjects included in this study underwent a CMR study using a 3.0 T clinical scanner (Skyra, Siemens). Cine images were obtained using a balanced steady-state free precession (SSFP) sequence in combination with parallel imaging (SENSitivity Encoding, factor 2) and retrospective gating during expiratory breath- hold (TE/TR/flip-angle: 1.7 ms/3.4 ms/60°, spatial resolution 1.8 × 1.8 × 8 mm, temporal resolution of 25 frames/cycle). Routine CMR analysis of cardiac volumes, function and mass was performed using commercially available software Medis Suite MR v2.1 (Medis medical imaging systems, Leiden, The Netherlands) [15] using a stack of gapless short axis (SAX) cine slices. Left ventricular endocardial borders were drawn manually at end-diastole and end-systole. The papillary muscles were traced and included as part of the LV cavity volume. Left ventricular end-diastolic (EDV) and end-systolic (ESV) volumes were determined using Simpson’ s rule. Ejection fraction (EF) was computed as EDV-ESV/EDV. All volumetric indices were normalized to body surface area (BSA).

Single cine slice long-axis views (LAX, 2-, 3-, and 4 chamber view) were used for GLS analysis using CMR-FT. Images were analysed off-line using two commercially available software packages: Medis Suite MR v2.1 (Medis medical imaging systems, Leiden, The Netherlands) and CVI42 Version 5.6.6 (Circle Cardiovascular Imaging Inc., Calgary, Canada). GLS was calculated as the average of the 3 LAX views and expressed as an absolute global peak systolic strain.

### Optimization and derivation of standardized postprocessing

All readers involved in this project had extensive previous experience of 2D speckle tracking by echocardiography. After training of vendor recommended postprocessing approaches (handbooks, website information, webinars, training with application specialists), a series of stepwise adjustments was examined by way of trial and error; these steps were implemented if shown to beneficially reduce the intra/inter-observer bias. Subsequently, datasets were analysed separately by two independent observers, resulting in three sets per each software or six outputs for each subject and implementation series. The readers were blind to their own, each other’s as well as previous measurements. Intraobserver measurements were repeated after an interim interval of 4 weeks.

**Fig. 1:**
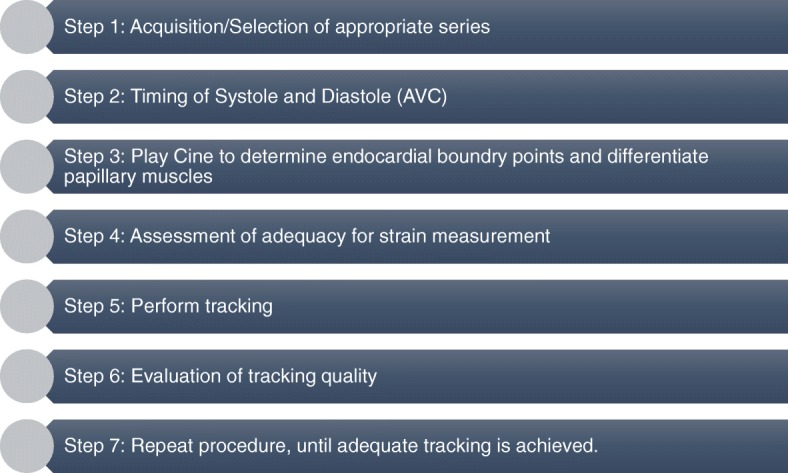
Steps for CMR-Feature tracking

**Fig. 2:**
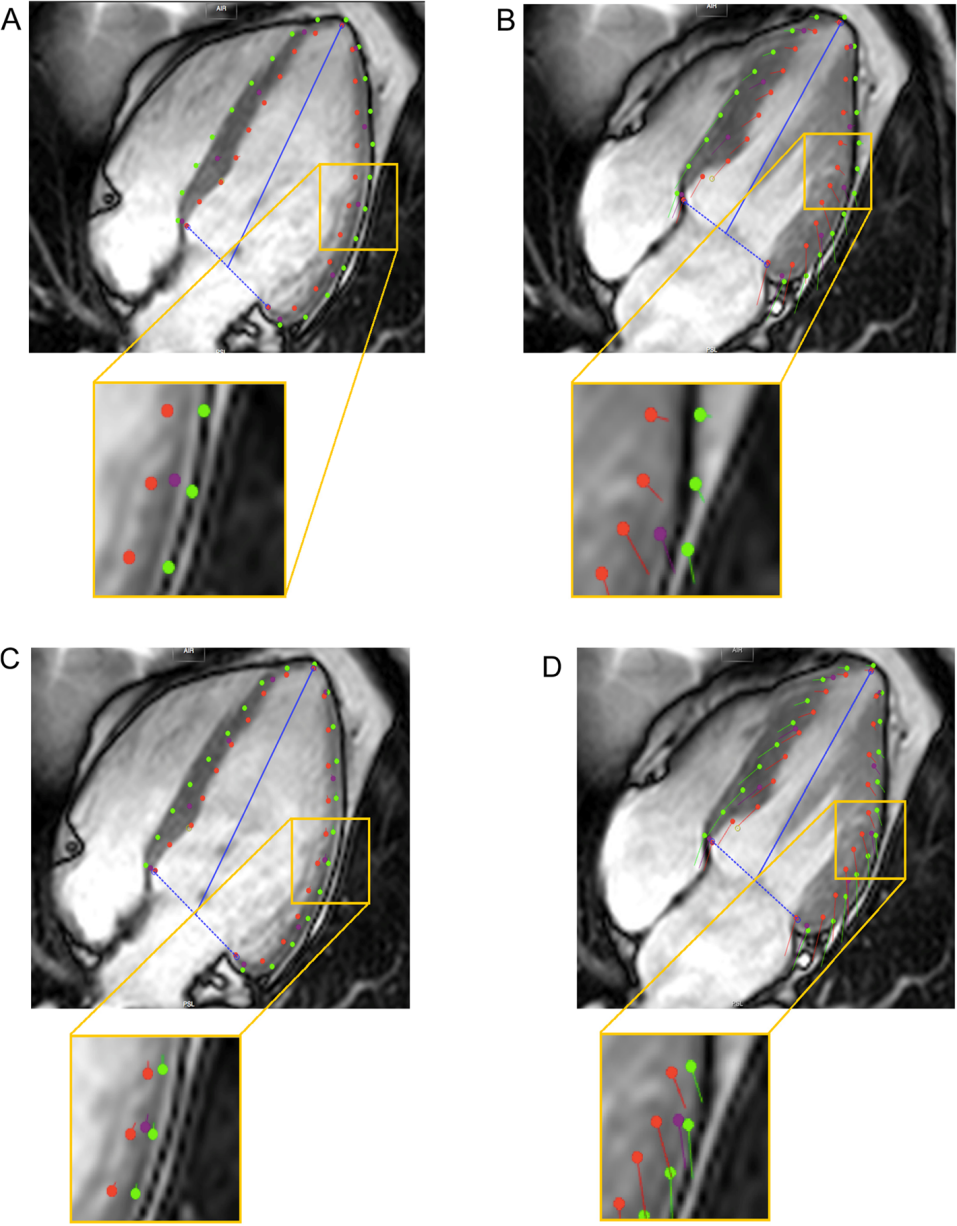
Examples for tracking problems and solutions. **a** - in this case, boundary points have been placed in the pericardium accidentally. **b** shows that there is no movement of the lateral wall boundary points in correlation to the ventricular contraction. **c** and **d**: In this case, boundary points were placed correctly and lateral wall tracking was improved

Optimization steps were based on a modified approach described previously [11], as well as comparative evaluation of both readers’ tracking results (Fig. 1). Common tracking errors and systematic differences were identified, and agreements were drawn and tested to support a reproducible approach for assessing GLS. Vendor-specific approaches to contour-manipulation were necessary. Using CVI-42, the epicardial and endocardial contours were manually delineated in all analysed sections with initial contours set at end diastole. The epicardial contours were purposefully placed slightly inside the myocardium avoiding at the epicardial border to reduce a common tracking failure resulting from placing the contours onto the boundary points of the pericardium (Fig. 2). Similarly, when placing the endocardial contours papillary muscles were avoided. When using Medis, the delineation of endocardial contours in all analysed sections was set at end systole, whereas epicardial contours (as well as all end-diastolic contours) were generated automatically. In most instances, the epicardial required manipulation by the observer, similar to the steps, described above. Care was taken to place the epicardial contour slightly within the myocardium to avoid tracking of the pericardium. Papillary muscles were excluded when placing endocardial contour. After completing the automated tracking process, the overall quality of contour placement was re-evaluated by the respective observer. Inadequate tracking, defined as apparent deviations of the contours from the endocardial and epicardial borders based on visual assessment, the contours were manually corrected, and the automated algorithm was reapplied (up to a maximum of 2 runs). Segmental regions of interest (ROIs), which persistently tracked poorly, were excluded from analysis. If persistent poor tracking included more than two segments in a single view, this patient’s case was excluded from the subsequent analysis.

### Statistical analysis

Statistical analysis was performed using SPSS, version 24. Normality of distributions were tested using Shapiro-Wilk test. Categorical data are expressed as counts (percentages), and continuous variables as mean ± SD or median (range), as appropriate. Mean difference (MD) ± SD was calculated from each group substracting the measured values of two different observers divided by the number of subjects measured in the respective group. Comparisons between groups were performed using Student t-test or one-way ANOVA for normally distributed variables, and chi2 and Mann-Whitney test for non-normally distributed variables. Fischer’s exact tests were used to compare proportions. Inter- and intra-observer variability was computed using the intra-class correlation coefficients (ICC) using a two-way mixed model with absolute agreement between measures and coefficient of variance (CV) and the Bland-Altman plots. Effect size between controls and patients based on GLS was assessed Cohen’s D as well as using receiver operating characteristic (ROC) curve analysis and calculation of the area under the curve (AUC); AUCs pre and after standardisation were compared using z-test statistics. Cohen’s D were calculated comparing the mean GLS values and the standard deviations pre- and poststandardization with the respective control group. The AUCs were calculated by integration of the graph produced by drawing the sensitivity of GLS on the y-axis and 1-specificity on the x-axis in terms of discriminating between health and disease. All tests were two-tailed and *p*-values <0.05 were considered statistically significant.

## Results

**Fig. 3:**
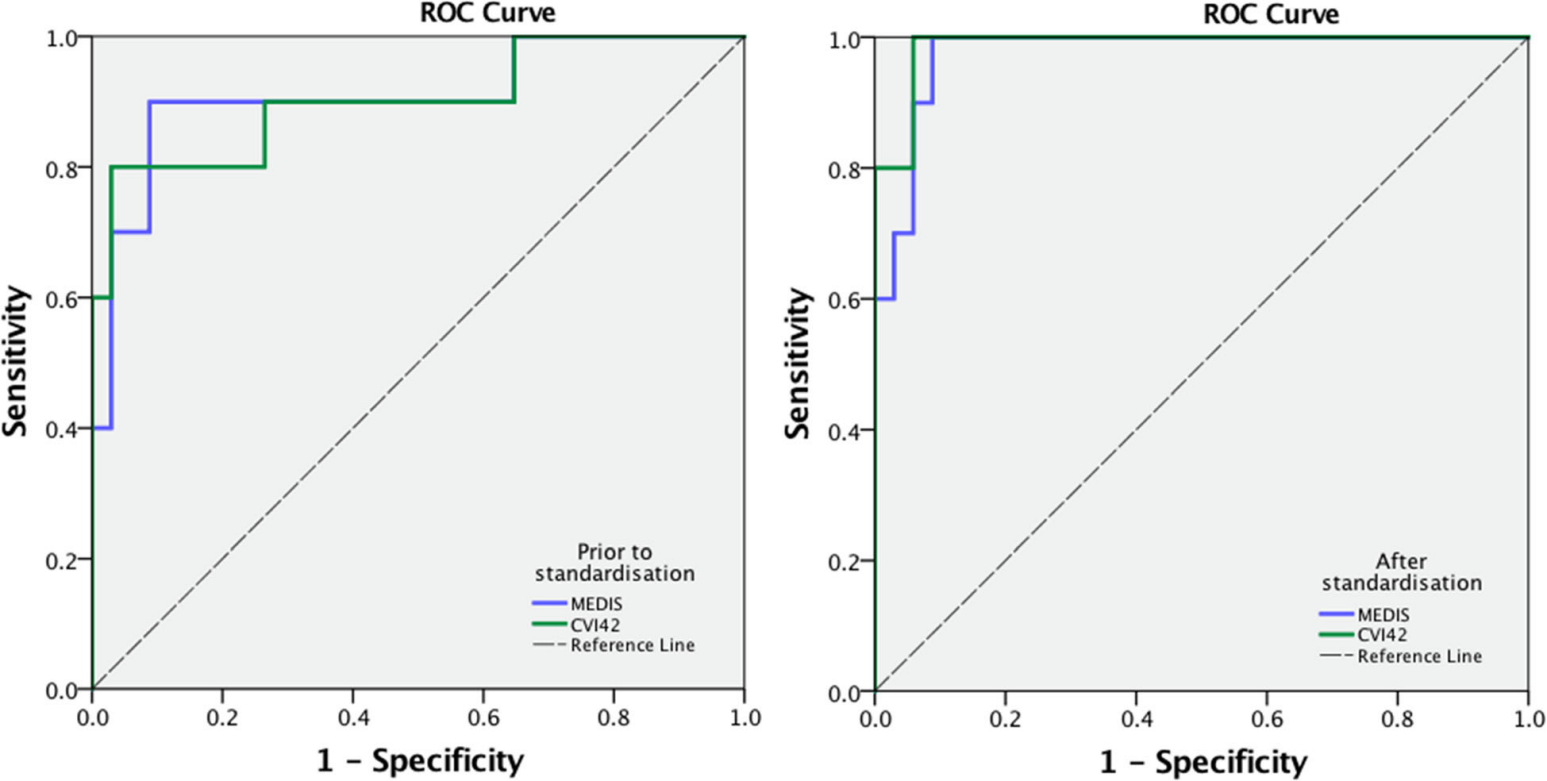
ROC Curve analyses for discrimination between health vs disease

**Fig. 4:**
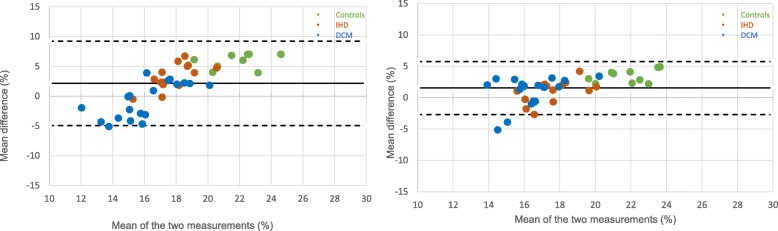
Results of reproducibility for intervendor agreement between Medis and CVI42. **a** – Bland Altman pre-standardization (Mean = 2.14; limits of agreement: +1.96s = 9.21 -1.96s = -4.33), (**b**) - Bland Altman post-standardization (Mean = 1.54; limits of agreement: +1.96s = 5.76 -1.96s = -2.69)

**Fig. 5:**
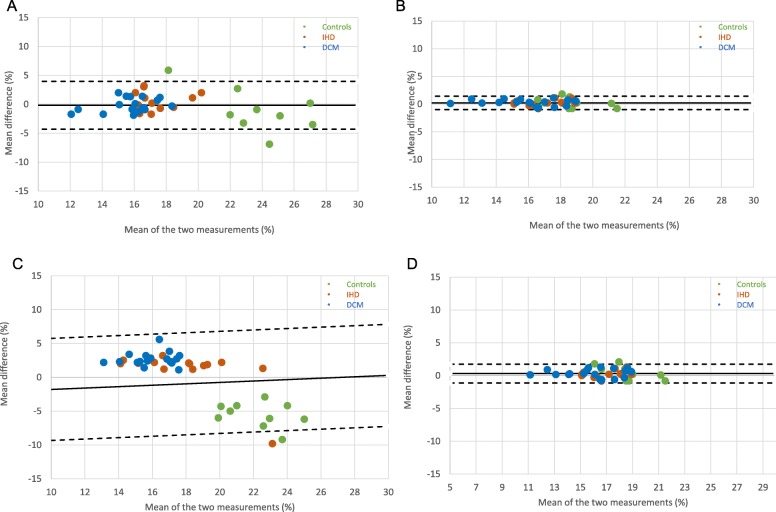
Bland Altman plot for observer agreement Medis: **a**- pre-standardization intraobserver (Mean = -0.18; limits of agreement: +1.96s = 3.96 -1.96s = -4.32); **b**- post-standardization intraobserver (Mean = 0.23; limits of agreement: +1.96s = 1.44 -1.96s = -0.98); **c** -pre-standardization interobserver (Mean = 0.29; limits of agreement: +1.96s = 7.84 -1.96s = -7.25); **d**- post-standardization interobserver (Mean = 0.33; limits of agreement: +1.96s = 1.76 -1.96s = -1.10)

**Fig. 6:**
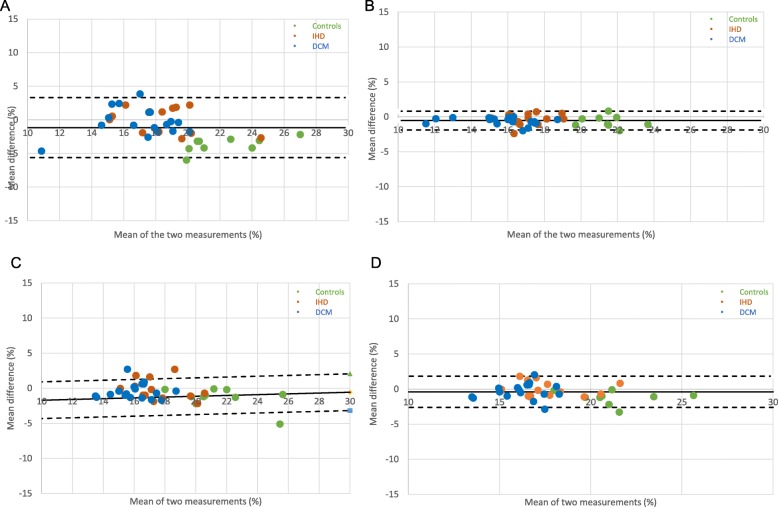
Bland Altman plot for observer agreement CVI42. **a** pre-standardization intraobserver (Mean = -1.16; limits of agreement: +1.96s = 3.30 -1.96s = -5.63); **b** post-standardization intraobserver (Mean = -0.54; limits of agreement: +1.96s = 0.80 -1.96s = -1.89); **c** pre-standardization interobserver (Mean = -0.57; limits of agreement: +1.96s = 2.05 -1.96s = -3.18); **d** post-standardization interobserver (Mean = -0.39; limits of agreement: +1.96s = 1.83 -1.96s = -2.60)

Baseline characteristics are displayed in Table 1. The final analysis included 44 subject cases: 15 patients with IHD and regional wall motion abnormalities, 19 patients with NIDCM and 10 healthy controls. Quantitative analysis was not diagnostic in 12 subjects, whereas in 17(39%) subjects 1 ROI had to be discarded. The results of GLS measurements before and after standardization are provided in Table 2 for both vendors. There were significant differences between controls and all patients, as well as patient subgroups, in most CMR measurements, as well as GLS (*p* < 0.005) (summarised in Table 2); these differences were accentuated through standardization procedures, as shown by significant increase in Cohen D’s and AUCs (Fig. 3, Table 3). The benefit of standardization was also evident through improved CV and ICC agreements between observers and the different vendors. Results of Bland Altman analyses for inter-and intraobserver reproducibility for both vendors prior to and after standardization are displayed in Table 2 (plots Figs. 4, 5 and 6). Limits of agreement and the coefficient of variation were reduced after standardization within each vendor, albeit more strongly for interobserver than intraobserver agreement (*p* = 0.03 vs <0.001, respectively) and more for MEDIS than CVI42 (Medis: *p* < 0.000; CVI42: *p* < 0.028).

**Table 1:** Subject characteristics

Variables	Controls (*n* = 10)	IHD (*n* = 15)	DCM (*n* = 19)
Males (n, %)	7 (70)	10 (67)	9 (60)
Age (years)	34 ± 11	59 ± 14*	52 ± 18*
Heart rate (bpm)	62 ± 8	64 ± 11	62 ± 9
BP systolic (mmHg)	119 ± 9	136 ± 13	129 ± 21
BP diastolic (mmHg	69 ± 8	82 ± 9	79 ± 8
BMI kg/m2	27 ± 9	29 ± 11	28 ± 9
LV-EDV (index), ml/m^2^	79 ± 16	78 ± 14	101 ± 11*§
LV-ESV (index) ml/m^2^	34 ± 7	47 ± 12*	56 ± 14*§
LV-EF (%)	57 ± 2	40 ± 10*	43 ± 7*
LV mass (index), g/m^2^	54 ± 11	46 ± 10	78 ± 9*
LGE (n, %)	/	15(100)	7 (47)
GLS (Medis)	20.12 ± 2.2	18.3 ± 2.2*	15.9 ± 2.4*§
GLS (CVI42)	23.51 ± 2.8	20.3 ± 3.9	16.8 ± 2.2

*IHD* Ischaemic heart disease, *DCM* Dilated cardiomyopathy, *BMI* Body mass index, *LV-EDV* Left ventricular end-diastolic volume, *LV-ESV* End-systolic volume, *GLS* Global longitudinal strain, one-way ANOVA, Bonferroni post-hoc tests for the differences from control group, * - for the differences from controls, § between the groups; *p* < 0.05 is considered significant

**Table 2:** Results of pre and post-standardization analyses

All subjects	Pre-Standardization		Post-Standardization		Sig. (*p*-value)	
GLS Medis						
Mean ± SD	16.9 ± 5.5		17.4 ± 2.7		0.32	
	IntraOB	InterOB	IntraOB	InterOB		
MD ± SD	−0.3 ± 2.1	0.1 ± 1.1	0.23 ± 0.7	−0.51 ± 1.9		
CV (%)	7.2	11	3.1	5.2		
ICC	0.979	0.914	0.996	0.98		
GLS CVI42						
Mean ± SD	19.0 ± 4.5		19.0 ± 3.9		0.98	
	IntraOB	InterOB	IntraOB	InterOB		
MD ± SD	−0.7 ± 2.3	−0.3 ± 1.3	− 0.2 ± 0.7	− 0.3 ± 1.1		
CV (%)	4.6	4.7	4.3	4.4		
ICC	0.978	0.964	0.995	0.993		
Controls	Pre-Standardization		Post-Standardization		Sig. (*p*-value)	
GLS Medis						
Mean ± SD	19.8 ± 5.3		20.71 ± 2.21		0.24	
	IntraOB	InterOB	IntraOB	InterOB		
MD ± SD	−0.9 ± 3.5	−0.4 ± 2.6	0.5 ± 1.1	0.51 ± 1.1		
CV (%)	3.8	6.5	2.2	2.2		
ICC	0.88	0.85	0.96	0.92		
GLS CVI42						
Mean ± SD	24.1 ± 5.2		23.51 ± 2.8		0.58	
	IntraOB	InterOB	IntraOB	InterOB		
MD ± SD	−0.7 ± 2.4	−0.6 ± 3.5	−0.7 ± 1.1	−1.2 ± 2.4		
CV (%)	3.4	5.8	1.6	2.0		
ICC	0.85	0.79	0.96	0.93		
IHD	Pre-Standardization		Post-Standardization		Sig. (*p*-value)	
GLS Medis						
Mean ± SD	16.2 ± 4.4		18.3 ± 2.2		0.45	
Cohen D	0.74		1.09			
	IntraOB	InterOB	IntraOB	InterOB		
MD ± SD	−0.4 ± 2.7	0.3 ± 1.9	0.3 ± 0.7	0.3 ± 0.8		
CV (%)	6.8	7.6	2.3	3.2		
ICC	0.79	0.81	0.92	0.89		
GLS CVI42						
Mean ± SD	19.4 ± 5.31		20.3 ± 3.9		0.63	
Cohen D	0.89		0.95			
	IntraOB	InterOB	IntraOB	InterOB		
MD ± SD	−0.8 ± 2.8	−0.5 ± 1.8	−0.4 ± 0.7	−0.5 ± 1.2		
CV (%)	3.5	3.6	1.8	2.4		
ICC	0.80	0.78	0.95	0.90		
DCM	Pre-Standardization		Post-Standardization		Sig. (*p*-value)	
GLS Medis						
Mean ± SD	16.38 ± 3.42		15.9 ± 2.4		0.599	
Cohen D	0.77		2.09			
	IntraOB	InterOB	IntraOB	InterOB		
MD ± SD	−0.2 ± 1.2	−0.9 ± 3.2	0.3 ± 0.7	−0.8 ± 0.6		
CV (%)	6	3.5	2.3	1		
ICC	0.74	0.79	0.93	0.96		
GLS CVI42						
Mean ± SD	17.7 ± 5.71		16.8 ± 2.2		0.26	
Cohen D	1.17		2.67			
	IntraOB	InterOB	IntraOB	InterOB		
MD ± SD	−0.6 ± 2.1	−0.3 ± 1.1	−0.5 ± 0.6	−0.4 ± 0.9		
CV (%)	3.5	3.6	1.3	2.3		
ICC	0.78	0.75	0.95	0.93		

*IHD* Ischaemic heart disease, *DCM* Dilated cardiomyopathy, *GLS* Global longitudinal strain, *MD* Mean differences, *SD* Standard deviation, paired and unpaired t-test for repeated measurements and the differences between the group, *p < 0.05 is considered significant

**Table 3:** Results of ROC analyses for separation between the groups prior to and after standardization (AUC, 95%CI)

Tests	AUC (95%CI)	
	Prestandardization	Poststandardization
MEDIS		
Controls vs All	0.909 (0.78–1.00)	0.976 (0.94–1.00)
Controls vs IHD	0.933 (0.82–1.00)	0.953 (0.88–1.00)
Controls vs DCM	0.89 (0.74–1.00)	0.985 (0.967–1.00)
CVI42		
Controls vs All	0.903 (0.77–1.00)	0.988 (0.96–1.00)
Controls vs IHD	0.867 (0.71–1.00)	0.958 (0.89–1.00)
Controls vs DCM	0.932 (0.82–1.00)	0.988 (0.94–1.00)

*IHD* Ischaemic heart disease, *DCM* Dilated cardiomyopathy

The common sources of poor reproducibility included:

- mis-selection of images (e.g. in case of repeated acquisitions due to artefacts, poor breath-holding, arrhythmia);
- the definition of end-diastole and end-systole (improved by visual determination of frames with biggest/smallest volume and closed heart valves);
- the placement of endo- and epicardial contours by
  - avoiding the dark pericardial rim or effusion (poor tracking);
  - exclusion of papillary muscles
  - re-evaluation of sufficient tracking.

Mean GLS values for all subjects were on average 2 percentage points different between the two vendors (*p* = 0.023), also on the subgroup level (*p* < 0.05), despite relatively high agreement in overall measurements (*r* = 0.85, *p* < 0.001). Although the inter-vendor difference persisted post-standardization, it was smaller compared to initial results, substantiating the fixed element of systematic difference in derived measurements with two vendors.

## Discussion

Results of our study reveal that post-processing approach to GLS matters with respect to the reproducibility of measurements and detection of effective difference in GLS between controls and patients. We demonstrate that standardization of GLS post-processing helps to reduce the random component of variability, introduced by inconsistencies between and within observers, while fixed systematic inter-vendor bias due to vendor related differences in image processing algorithms remained. We further show that the greater precision of measurements affords greater effect size, and as thus, improved discrimination between controls and subgroups of patients, which was not vendor-dependent. Results of our study provide a proof of concept that standardization of GLS measurements is an essential step in ensuring the reliable quantification of myocardial deformation, between different observers, users and across vendors.

Previous studies leading up to the present work have highlighted the differing normal values as well as the results for intra- and interobserver reproducibility, as well as between vendors and centres (summarised in Table 1S from PMID:19789193) [16]. In summary, the reported intraobserver CV ranges between 5.2–12.3%, whereas interobserver CV 10.9–15.4%, and our initial results agree well with these previous reports. However, we have shown that by following the standardization protocol considerably improves reproducibility of measurements, in the study group as a whole, as well as subgroups. By employing a standardization protocol, CMR-FT can become an objective and reproducible method for the quantification of LV deformation. Whereas the burden of contour manipulation may at first appear substantial, we have narrowed this down to a few essential and systematic steps, predictable of failure of tracking, as well as vendor-specific contour placement, which has proactively served to considerable improvement. This information is important as it may guide the necessary optimization steps of CMR-FT softwares, in order to adequately serve the clinical routine. Diversity of normal values is often noted limitation of CMR-FT, yet the range of the thus-far reported mean values is admittedly narrow (19–21.3%, Table 1S), also reproduced by the recent metanalysis by Vo et al., 20.1% [11]. Majority of the previous studies used TomTec based software [6, 7], a product using the same tracking algorithm as the MEDIS software, and our MEDIS derived values in controls reproduce these previous reports. The systematically higher measurements with CVI42 signal a very different image-processing approach; yet the high inter-vendor agreement of measurements suggests that although the softwares may be employing different algorithms, they track similar features of myocardial deformation. The benefits of standardization can further be seen through marked improvement of CV and ICC and Bland Altman plots, reflecting the effect of harmonization for both intra- and inter-observer variability. Reduction of the mean differences and limits of agreement translate into smaller dispersion of the GLS measurements, which is greater for interobserver reproducibility. Applying the standardized steps improved results for both vendors; vendor-specific steps clearly helped to reduce intervendor bias, again communicating a random variability component or, in other words, the many ways in which different observers could potentially use the different softwares. Our findings emphasize the role of clear and documented instructions and their unconditional implementation, in support of multi-user transferability in routine clinical practice.

The detection of early disease relies on precision in the technique, which can control for misclassification from healthy subjects. The patients and groups in our study were selected to be representative of the common clinical scenarios, where employment of GLS is known to be complementary to the assessment of global LV function, e.g. the mid-range LV-EF 30–50% [17]. Compared to controls, both patient groups were older and had significantly but similarly reduced global systolic function. In both groups, GLS values were significantly lower in comparison to controls. Of note, comparative GLS measurements between IHD and DCM group revealed significantly lower GLS in the DCM group (*p* < 0.001). This is an important observation, which is in part explained by considerably higher LV volumes in DCM group, indicating the presence of global remodelling and consequently, operation at much higher loading. In the IHD group, GLS is reduced considerably, but only owing to severe regional impairment, whereas the preserved myocardium at first compensates with hypertrophic response and not change of loading [1, 18]. Given the rather homogenous presentation of cases within the model disease groups, the AUC for separation of patient groups from healthy controls were excellent before and after standardization, although additional improvement remains notable.

The introduction of CMR-FT was long hailed as a much-needed clinical application that reuses the routine cine acquisitions, while reducing the need for additional imaging that encode the changes with myocardial deformation, such as tagging. The overall viability of this technology appeared to depend on the availably of a quick, sleek and foremost accurate offline postprocessing, which resulted in offspring of several dedicated software products for CMR-FT. Yet the results of CMR-FT analyses vary from vendor to vendor and remain highly observer dependent. Several solutions were proposed, foremost the averaging of results of repeated analyses for increasing intra-vendor reproducibility [11]. Our results reveal that benefit of such solution is likely dubious, the doubling or tripling of analysis time notwithstanding, as the source of high variability primarily arise from the tracking failure of automatically detected (auto-) contours, which cannot be improved by repetition, but manipulation of contour placement on post-processed SSFP images. In our study, this approach turned out to influence most strongly the accuracy of CMR-FT and several reasons underlie this observation. There are many independent variables that cannot be improved by repetitive tracking including image quality, frame rate, slice geometry (e.g. cutting through the papillary muscles), observer and centre experience. Image quality will suffer with poor breath-capacity, mitral annular calcification, pericardial effusion, mis-triggering, low frame rate and imperfect slice positioning, and will result in poor auto-tracking due to difficult endo- and epicardial border definition and misallocation of placed boundary points. Institutional structures mandating standardised approaches and providing adequate training will have high impact on reproducibility and precision.

## Conclusion

Standardization of GLS postprocessing helps to reduce the random component of variability, introduced by inconsistencies of and between observers, and to some extent also intervendor variability. There remains fixed systematic inter-vendor bias due to vendor related differences in image processing algorithms. Greater precision of measurements affords an improved effect size, and as thus, discrimination between controls and subgroups of patients, irrespective of the choice of postprocessing software or underlying pathophysiology. Results of our study provide a proof of concept that standardization of GLS measurements is an essential step in ensuring the reliable quantification of myocardial deformation, between different observers, users and across vendors, and for implementation of CMR-FT in clinical routine.


Additional file 1: Video GLS.


## Data Availability

Relevant data including subject characteristics, pre- and poststandardization results of strain analyses and the results of statistical analyses are provided in Tables 1, 2 and 3. For more information and access to all analysed datasets please contact the corresponding author. We are able to share the complete datasets if required.

## Abbreviations

AUC: Area under the curve
BMI: Body mass index
BP: Blood pressure
BSA: Body surface area
CMR: Cardiac magnetic resonance
CMR-FT: Cardiac magnetic resonance feature tracking
SSFP: steady-state free precession
CV: Coefficient of variation
DCM: Dilated cardiomyopathy
EDV: End diastolic volume
EF: Ejection fraction
ESV: End systolic volume
GLS: Global longitudinal strain
ICC: Intra-class correlation coefficient
IHD: Ischaemic heart disease
LAX: Long axis
LGE: Late gadolinium enhancement
LV: Left ventricle
NIDCM: Non-ischaemic dilated cardiomyopathy
ROC: Receiver operated characteristic
ROI: Region of interest
RWMA: Regional wall motion abnormalities
SAX: Short axis
